# Modern Diagnostic Modalities for Fuchs’ Endothelial Corneal Dystrophy: A Comparative Analysis Using Scheimpflug Tomography

**DOI:** 10.3390/medicina62071309

**Published:** 2026-07-06

**Authors:** Vladislava Yotsova, Mladena Radeva, Valeri Sheherov, Zornitsa Zlatarova

**Affiliations:** Department of Ophthalmology and Visual Sciences, Faculty of Medicine, Medical University of Varna, 55 Marin Drinov, 9000 Varna, Bulgaria; vladka7@mail.bg (V.Y.); dr.sheherov@gmail.com (V.S.); zizlatarova@gmail.com (Z.Z.)

**Keywords:** fuchs dystrophy, Scheimpflug tomography, corneal dystrophy

## Abstract

*Background and Objectives:* Fuchs’ endothelial corneal dystrophy (FECD) is a progressive disorder characterized by endothelial cell loss, corneal edema, and reduced transparency. Scheimpflug tomography enables objective evaluation of the corneal structure, including densitometry as a marker of optical quality. This study aimed to assess topographic and microstructural corneal parameters in FECD patients using Pentacam tomography and to evaluate their diagnostic utility. *Materials and Methods:* A total of 89 subjects (178 eyes) were included: 47 patients with FECD (94 eyes) and 42 healthy controls (84 eyes). Participants were stratified by age and sex. All underwent comprehensive ophthalmic examination and corneal imaging with Pentacam HR. Corneal densitometry was analyzed in four concentric zones (0–2, 2–6, 6–10, and 10–12 mm) and three layers (anterior, central, posterior). Statistical analysis was performed using SPSS v.19, with *p* < 0.05 considered significant. *Results:* Densitometry values increased with age in both groups, with significantly higher values in FECD patients, particularly in peripheral zones (6–12 mm). The highest backscatter was consistently observed in the anterior corneal layer. Significant differences between FECD and controls were found in specific age subgroups and corneal regions. A progressive increase in backscatter from Descemet’s membrane was observed, corresponding to a transition in densitogram patterns from a “high-backed chair” to a “hammock” configuration. Disease progression appeared more pronounced in male patients. *Conclusions:* Corneal densitometry obtained by Scheimpflug tomography provides reliable quantitative and qualitative indicators of FECD progression. Its combined use with topographic parameters enhances early diagnosis and disease monitoring.

## 1. Introduction

Fuchs’ endothelial corneal dystrophy (FECD) is a bilateral, asymmetric, slowly progressive degenerative disease affecting the corneal endothelium, leading to reduced endothelial cell count, impaired barrier and pump functions, corneal hydration, and eventual loss of transparency [1,2]. It affects approximately 4–7% of the population, predominantly females over 40–50 years, and is a leading cause (36%) of corneal transplantation in the United States [3]. Pathogenesis involves autosomal dominant inheritance with variable expressivity, linked to mutations in genes such as *COL8A2*, *TCF4*, *TCF8*, *SLC4A11*, and *AGBL1*, rendering cells susceptible to oxidative stress and apoptosis [4]. Clinical manifestations include central guttae (collagen deposits on Descemet’s membrane), stromal edema, epithelial bullae, fibrosis, and neovascularization in advanced stages [5]. Diagnosis relies on clinical history and examination, supplemented by imaging modalities like specular microscopy for endothelial morphology and Scheimpflug tomography for thickness, densitometry, and topographic features. Recent studies have emphasized the role of Scheimpflug imaging and densitometry as reproducible non-invasive biomarkers for FECD staging and follow-up, particularly when combined with pachymetric and tomographic indicators.

This study aimed to evaluate whether Scheimpflug-derived corneal densitometry parameters differ between FECD patients and healthy controls across age and sex subgroups and to determine their potential utility as quantitative biomarkers for disease progression and monitoring.

## 2. Materials and Methods

1. The study was approved by the Ethics Committee of Medical University-Varna (Protocol No. 130/20.04.2023) and adhered to the Declaration of Helsinki. All participants provided informed consent.
2. **Participants** The study was conducted from May 2023 to December 2024 and included 89 individuals: 58 females (65.17%) and 31 males (34.83%). Groups were Control (42 individuals, 84 eyes, no FECD) and FECD (47 individuals, 94 eyes). Subgroups were divided by 10-year age intervals: females (50–59, 60–69, 70–79, 80–89 years); males (60–69, 70–79, 80–89 years).
3. Inclusion criteria for controls: healthy volunteers >18 years with informed consent. Exclusion: FECD, intraocular inflammation, glaucoma, prior surgery, or no consent.
4. For FECD: patients >18 years with FECD, informed consent, no other ocular/systemic diseases. Exclusion: no FECD, other ophthalmologic conditions, <18 years, inflammation, prior surgery, mental impairments, or no consent.
5. **Clinical Examination** Medical and family history were recorded via a customized questionnaire assessing visual quality and anterior segment discomfort. Ophthalmic evaluation included best-corrected visual acuity (BCVA), intraocular pressure (non-contact tonometer, Oculus Corvis ST, Oculus Optikgeräte GmbH, Wetzlar, Germany), anterior segment biomicroscopy (Haag-Streit, AG, Köniz, Switzerland), and fundus stereo-ophthalmoscopy (+90D lens post-mydriasis with tropicamide).

**Corneal Tomography**

Corneal tomography (Pentacam HR; Oculus Optikgeräte GmbH, Wetzlar, Germany) was performed according to a previously described protocol. Only images of acceptable quality were included in the study.

The examination was performed in a non-contact manner, with each eye being measured three times. After completion of the examination, the device performed an automatic analysis. The software of the Pentacam Corneal Topographer (version 6.10r56) generates color maps of the cornea—topographic, pachymetric, and anterior chamber depth. It generates and reproduces a map showing quantitative light backscatter, called a densitogram or densitometric map [6].

**Densitometry**

Corneal densitometry is a measure of light backscatter and an objective measurement of the optical density of the cornea. The measurement is part of the standard software of the Pentacam corneal tomograph. The measurement protocol takes a series of 25 images (1003 × 520 pixels) along different meridians with a uniform blue light source. During the analysis, the program automatically determines the location of the corneal apex and analyzes an area with a diameter of 12 mm around it. The output data are expressed in gray scale units (GSU). According to this scale, the software determines the minimum light scattering with 0 (maximum transparency) and the maximum scattering with 100 (minimum transparency). The cornea is software divided into 4 concentric radial zones (first, central zone—with a diameter of 2 mm; second—a ring from 2 mm to 6 mm; third—from 6 mm to 10 mm; fourth—from 10 mm to 12 mm). The cornea can also be divided into an anterior layer, which includes the anterior 120 µm, and a posterior layer—the posterior 60 µm. The central layer does not have a fixed thickness but is determined by subtracting the two known layers from the total corneal thickness (23, 24). Densitometry has been introduced into practice to quantify corneal transparency as an optical index of corneal health since backscatter of light in a normal cornea is minimal. Corneal backscatter intensity is used to assess various disease states that result in changes in the water content, collagen fiber diameter, and abnormal macromolecule accumulation, which reduces corneal transparency and impairs backscatter. In a healthy patient, the densitogram shows a single anterior spiky hump resulting from epithelial backscatter, with central flattening and smoothing of the second hump, presenting a “high-backed chair” pattern. In patients with advanced FECD, a typical densitogram finding is a “hanging hammock” pattern. It shows two spiky humps with a central depression that appear like a “hanging hammock.” The first hump corresponds to epithelial backscatter, and the second to backscatter resulting from damaged Descemet’s membrane [7].

**Statistical Methods**

Both eyes from individual participants were included because FECD is typically bilateral; however, analyses were performed at the eye level with subgroup comparisons interpreted cautiously to reduce the risk of inter-eye correlation bias. No formal power calculation was performed because of the exploratory observational design of the study.

Data processing was performed with the SPSS version 19 statistical package. A significance level of α = 0.05 was selected, i.e., all values with *p* < 0.05 were considered statistically significant. Ninety-five percent confidence intervals were calculated to assess the values in the population. The obtained data are presented in graphical and tabular form.

For analysis and interpretation of the data in order to reveal the essence of the observed phenomena, the subject of this study, we used:

Descriptive analysis:

- Arithmetic mean, median measures for assessing central tendency;
- Minimum and maximum value;
- Standard deviation (SD)—measure for assessing dispersion.

Independent samples *t*-test—applied to variables with a normal distribution.

Paired samples *t*-test—applied to comparing two related groups.

One-Way ANOVA—for comparing mean values between two or more groups.

## 3. Results

The distribution of the 89 subjects studied by basic demographic characteristics and health status is presented in Table 1.

The results of the descriptive analysis show that the average age of women with FECD is approximately 71 years with a deviation ±9.428 years and a median age of 72 years. The youngest age among women with FECD is 54 years, and the oldest is 85 years. The average age of men with FECD is approximately 75 years (75, 44 years) with a deviation ±6.928 years and a median age of 75, 50 years. The youngest age among men with FECD is 65 years, and the oldest is 88 years.

### 3.1. Evaluation of the Data Obtained from Pentacam Scheimpflug Tomography

Using the Pentacam HR; Oculus, 178 eyes of 89 patients with FECD and controls were examined. Patients from both groups were divided by gender and age, and the obtained results were compared. Information was extracted from the topographic maps regarding backward light scattering from the cornea, CCT, the location of the thinnest point of the cornea, and loss of regular isopachs.

### 3.2. Corneal Densitometry

With the help of the Pentacam Scheimpflug tomograph, we were able to quantitatively determine corneal opacity as an optical index for corneal health, since backward light scattering in a normal cornea is minimal. The software (version 6.10r56) divides the cornea into 4 concentric radial zones (the first, central zone—with a d 2 mm; second—ring from 2 mm to 6 mm; third—from 6 mm to 10 mm; fourth—from 10 mm to 12 mm) and into 3 layers (anterior layer—120 µm, posterior layer—60 µm, and central layer, which does not have a fixed thickness but is determined by subtracting the two known layers from the total thickness of the cornea).

We compared the obtained mean values in the different zones and layers between the various age subgroups and the respective controls. The results are presented in following tables (Table 2). Detailed numerical datasets are additionally provided in Supplementary Tables S1–S4.

In the 50–59 age subgroup, the mean corneal densitometry value for the entire 12 mm diameter zone was 20.138 + 2.2659 for FECD patients and 16.900 + 0.7071 for female controls, demonstrating statistical significance (*p* = 0.005). When considered by radial zones, in FECD patients and controls, densitometry values were lowest in the paracentral radial zone (15.725 + 0.9192 and 15.800 + 0.4243, respectively), followed by the central zone and highest in the periphery (28.363 + 4.5356 and 19.350 + 1.2021, respectively). No statistically significant difference was found between the densitometric values of the central 2 mm zone and the surrounding 2–6 mm ring (One-Way ANOVA, *p* = 0.165). The values for the 6–10 and 10–12 mm zones in patients with FECD were significantly higher compared to the other two zones (*p* < 0.001). When the cornea was divided into layers, the highest degree of backscatter was observed in the anterior layer (26.463 + 3.2802), with the value being significantly higher than the central and posterior layers (*p* < 0.001). The results obtained in the table show statistical significance in terms of backscatter in the central and posterior part of the 0–2 mm zone in favor of the controls (*p* = 0.005 and 0.040). In patients with FECD, a statistically significant increase in backscatter was found in the last 2 zones and in all layers compared to the controls (Table 3).

In the subgroup of women aged 60–69, the mean corneal densitometry value for the entire 12 mm diameter zone was 20.550 + 1.8509 for patients with FECD and 20.467 + 2.0146 for female controls, with no statistical significance. When considered by radial zones, in patients with FECD and controls, the densitometric mean values were lowest in the paracentral zone (2–6 mm) (16.188 + 1.9874 and 15.617 + 1.2828, respectively), followed by the central and highest in the periphery (28.675 + 3.5596 and 29.517 + 3.1815, respectively). There was no statistically significant difference between the densitometric values of the central 2 mm zone and the surrounding 2–6 mm ring (One-Way ANOVA, *p* = 0.083). The values for the 6–10 and 10–12 mm zones in patients with FECD were significantly higher than the other two zones (*p* < 0.001). The data were similar in control patients. Layer-wise, the highest degree of backscatter was observed in the anterior layer (26.525 + 3.3435), with the value being significantly higher than the central and posterior layers (*p* < 0.001). In controls, the backscatter was again higher in the anterior layer, but here *p* = 0.004 for the 6–10 mm zone and 0.001 for the 10–12 mm zone. The results in the table show a statistically significant increase in backscatter in the posterior part of the 0–2 mm zone (*p* < 0.001). In the radial zone, 6–10 mm, a significant difference was found in the posterior layer (*p* = 0.011). In the remaining zones and layers, no statistically significant differences were found between patients with FECD and controls (Table 4).

In the subgroup of women aged 70–79, the mean corneal densitometry value for the entire 12 mm diameter zone was 25.175 + 4.5714 for patients with FECD and 24.700 + 2.6005 for female controls, with no statistical significance between the two groups. When considered by radial zones, for patients with FECD and controls, the densitometric mean values were lowest in the central zone for patients with FECD (17.275 + 1.4017) and in the radial zone 2–6 mm for controls (16.662 + 1.4928) and highest in the periphery (35.063 + 4.7118 and 36.163 + 8.8880). There was no statistically significant difference between the densitometric values of the central 2 mm zone and the surrounding 2–6 mm ring (One-Way ANOVA, *p* = 0.547). The values for the 6–10 and 10–12 mm zones in patients with FECD were significantly higher than the other two zones (*p* < 0.001). The data were similar in control patients. When the densitometric values were divided by layer, the highest degree of backscatter was observed in the anterior layer (33.833 + 6.6833), with the value being significantly higher than the central and posterior layers (*p* < 0.001). In controls, this pattern was maintained. The results in the table show a statistically significant increase in backscatter in the posterior part of the 0–2 mm zone (*p* = 0.047). In the radial zone, 10–12 mm, a significant difference was found in the posterior layer (*p* < 0.001) in favor of the controls. In the remaining zones and layers, no statistically significant difference was found between patients with FECD and controls (Table 5).

In the subgroup of women aged 80–89, the mean corneal densitometry value for the entire 12 mm diameter zone was 24.057 + 4.2583 for patients with FECD and 22.660 + 4.8454 for female controls, with no statistical significance between the two groups. When considered by radial zones, in patients with FECD and controls, the densitometric mean values were lowest in the 2–6 mm zone (18.550 + 2.4140 and 16.290 + 1.3203, respectively), followed by the central, and highest in the periphery (30.586 + 8.0551 and 33.250 + 11.8778, respectively). There was no statistically significant difference between the densitometric values of the central 2 mm zone and the surrounding 2–6 mm ring. The values for the 6–10 and 10–12 mm zones in patients with FECD were significantly higher than the other two zones (*p* < 0.001). The data were similar in control patients. When the densitometry values were divided by layer, the highest degree of backscatter was observed in the anterior layer (32.271 + 6.0982), with the value being significantly higher than the central and posterior layers (*p* < 0.001). In controls, this pattern was preserved. The results obtained in the table show a statistically significant increase in backscatter in the 0–2 mm and 2–6 mm zones in all parameters except the posterior layer, although a significant progression was observed in the posterior layer in these zones in patients with FECD compared to controls. In 10–12 mm, a significant difference in backscatter in the anterior layer (*p* < 0.016) was established in favor of controls. In the remaining zones and layers, no statistically significant difference was found between patients with FECD and controls (Table 6).

We compared the values obtained from densitometry between patients with FECD from age subgroups 50–59 years and 80–89 years. As a test value, we used the average values of patients aged 50–59 years. In the central 2 mm zone, statistical significance of the parameter in the anterior and central zones was established. In radial zones 2–6 mm and 6–10 mm, a clinically significant increase in the parameter was observed. Regarding corneal densitometry for the entire zone with a diameter of 12 mm, it is seen that a statistically significant increase in backscatter of light was established in all three layers (Table 7).

In the age subgroup (men) 60–69 years, the mean value of corneal densitometry for the entire area with a diameter of 12 mm was 18.750 + 0.7895 for patients with FECD and 22.890 + 2.5826 for male controls, with statistical significance being demonstrated but in favor of controls. There was a significant increase in the backscatter of the entire cornea in male controls compared to those with FECD (*p* = 0.002). If we compare the same age subgroup of female patients with FECD and controls, a statistically significant difference was also found in this indicator (*p* = 0.020 and *p* = 0.016, respectively). When considered by radial zones, in FECD patients and controls, densitometric values were lowest in the paracentral radial zone (16.275 + 0.4031 and 16.040 + 1.2756, respectively), followed by the central zone and highest in the periphery (10–12 mm radial zone) (24.775 + 4.1210 and 34.450 + 6.6958, respectively), similar to women of the same age group. There was no statistically significant difference between the densitometric values of the central 2 mm zone and the surrounding 2–6 mm and 6–10 mm radial rings. The mean value for the 10–12 mm zone in FECD patients was statistically significantly higher compared to the central and paracentral zones (*p* = 0.038; *p* = 0.026) but not for the 6–10 mm radial zone (*p* = 0.064). In contrast to women of the same age subgroup, the backscatter of light in the two peripheral radial zones was statistically significantly higher than the central and paracentral zones. When the densitometry values were divided by layer, the highest degree of backscatter was observed in the anterior layer (24.425 + 1.0210), the value being significantly higher than the central (*p* = 0.001) and posterior layers (*p* < 0.001), while in the controls, *p* < 0.001 for the central and posterior layers. The results obtained in the table show statistical significance in terms of backscatter in all layers in the radial zones 6–10 mm and 10–12 mm of the zone 0–2 mm of the controls, while in the central and paracentral zones, no clinically significant difference was found between the two subgroups (Table 8).

In the subgroup of men aged 70–79, the mean corneal densitometry value for the entire 12 mm diameter area was 27.162 + 4.0415 for patients with FECD and 25.356 + 2.4895 for male controls, with no statistical significance between the two groups (*p* = 0.247). If we compare with women from the same age subgroup, in patients with FECD and controls, no statistically significant difference was found in this indicator (*p* = 0.207 and *p* = 0.452, respectively). When considered by radial zones, in FECD patients and controls, the densitometric mean values were lowest in the central zone for FECD patients (19.325 + 1.4400) and in the radial zone 2–6 mm for controls (17.856 + 1.4152) and highest in the zone 6–10 mm for FECD patients (34.313 + 8.6062) and the peripheral zone for controls (38.333 + 11.7546). In contrast to men, in women with FECD, the highest degree of backscatter was observed, similar to controls, in the peripheral 10–12 mm zone. There was no statistically significant difference between the densitometric values of the central 2 mm zone and the surrounding 2–6 mm ring (One-Way ANOVA, *p* = 0.154) in men with FECD. The values for the 6–10 and 10–12 mm zones in patients with FECD were significantly higher compared to the other two zones (for the 6–10 mm zone—*p* = 0.002 and *p* = 0.003, respectively compared to the central 0–2 mm and 2–6 mm zones; for the 10–12 mm zone *p* = 0.001). The data were similar in the control patients (*p* = 0.001). When the densitometry values were divided by layer, the highest degree of backscatter was observed in the anterior layer (37.788 + 6.7198), the value being significantly higher than the central (*p* = 0.001) and posterior layers (*p* < 0.001). In the controls, this pattern was preserved (*p* < 0.001). The results in the table show a statistically significant increase in backscatter in the central and posterior parts of the 0–2 mm zone (*p* = 0.001 and *p* = 0.004). In radial zone 6–10 mm, a statistically significant increase in backscatter was found in all layers. In radial zone 10–12 mm, a significant difference was found in the central layer (*p* = 0.020) in favor of the controls. In the remaining zones and layers, no statistically significant difference was found between patients with FECD and controls (Table 9).

In the subgroup of men aged 80–89, the mean corneal densitometry value for the entire 12 mm diameter area was 26.700 + 4.5233 for patients with FECD and 30.450 + 6.0973 for male controls, with no statistical significance between the two groups (*p* = 0.098). If we compare with women from the same age subgroup, in patients with FECD and controls, no statistically significant difference was found in this indicator (*p* = 0.212 and *p* = 0.084, respectively). When considered by radial zones, in patients with FECD and controls, densitometric mean values were lowest in the radial zone 2–6 mm (21.000 + 3.5777 and 22.750 + 7.3178, respectively) and highest in the peripheral zone 10–12 mm (33.717 + 3.2382 and 41.600 + 9.8718, respectively). Similar findings were found in women of the same age subgroup. There was no statistically significant difference between densitometric values of the central 2 mm zone and the surrounding 2–6 mm ring in men with FECD and controls. The values for the 6–10 and 10–12 mm zones in patients with FECD were significantly higher compared to the other two zones, being statistically significant for the 6–10 mm zone compared to the 2–6 mm zone (*p* = 0.015) and for the 10–12 mm zone compared to the central and paracentral 2–6 mm zones (*p* = 0.001 and *p* < 0.001, respectively). In controls, the values were also statistically significantly increased (for the 6–10 mm zone *p* = 0.16 and *p* = 0.13, and for the 10–12 mm zone *p* = 0.36 and *p* = 0.032, respectively, compared to the central 0–2 mm and radial 2–6 mm zones). When densitometry values were divided by layer, the highest degree of backscatter was observed in the anterior layer (37.600 + 7.0759), with the value being significantly higher than the central (*p* = 0.005) and posterior layers (*p* = 0.001). In the controls, this pattern was violated, with *p* = 0.079 when compared to the central layer and *p* = 0.030 when compared to the posterior layer. The results obtained in the table show a statistically significant increase in backscatter in the anterior and central part of the 6–10 mm zone (*p* = 0.015 and *p* = 0.027) and the 10–12 mm zone (*p* = 0.004 and *p* = 0.001) in favor of the controls. We did not find a statistically significant increase in backscatter in the remaining layers and zones (Table 10).

In both female patients with FECD and male patients in age subgroups 60–69 and 80–89, we compared the values obtained from densitometry to determine the presence of progression in the degree of backscattering in the different layers and zones of the corneas. As a test value, we used the average values of patients aged 60–69. In the central 2 mm zone, statistical significance of the parameter was established in the anterior and posterior zones but not in the central part, although an increase in the degree of backscattering was also seen in it. In the remaining radial zones, we observed a clinically significant increase in the parameter, with the exception of the posterior part in radial zone 10–12 mm, where the value was borderline.

When comparing densitometry results in men and women in the 80–89 age subgroup, statistical significance was demonstrated only for the factor corneal backscatter in the most peripheral radial zone (10–12 mm) (*p* = 0.027).

When analyzing densitograms of women with FECD aged 50–59, it was found that 21.4% (3 eyes) of the examined eyes had a mild posterior spinous hump, corresponding to backscatter at the level of Descemet’s membrane. 78.6% (11 eyes) of the examined eyes showed a “high-backed chair” pattern on the densitogram.

In the analysis of densitograms of women with FECD aged 60–69, it was found that in 71.4% (10 eyes) of the examined eyes, a mild posterior spicule was observed, and 28.6% (4 eyes) of the examined eyes showed a “high-backed chair” pattern on the densitogram.

In the next age group (70–79 years) of women with FECD, densitograms of 18 eyes showed that in only 27.8% (5 eyes) of the examined eyes, the densitogram showed a “high-backed chair” pattern. The remaining 72.2% were conditionally divided into those with a lower posterior spicule, corresponding to that in the previous groups (38.9% of the examined eyes) and densitograms with more pronounced backscatter from Descemet’s membrane (33% of the examined eyes).

In the last age group (80–89 years) of women with FECD, densitograms of 16 eyes were analyzed. It was found that 6.25% (1 eye) had a “high-backed chair” pattern. Of the remaining 93.75%, 31.25% (5 eyes) had a low posterior spicule, and the remaining 50% had a strong backscatter from Descemet’s membrane, corresponding to a “hammock” pattern in the densitogram.

As can be seen from the results presented so far, with advancing age in patients with FECD, the backscatter of light from Descemet’s membrane gradually increased, and the densitogram gradually changed from a “high-backed chair” pattern to a “hammock” pattern.

In the analysis of densitograms of men with FECD aged 60–69 years, it was found that 25% (2 eyes) of the examined eyes showed a barely noticeable posterior spinous hump, corresponding to backscatter at the level of Descemet’s membrane, and 75% (6 eyes) of the examined eyes showed a “high-backed chair” pattern on the densitogram.

In the subgroup of men with FECD aged 70–79 years, from the densitograms of 16 eyes, it was found that only 12.5% (2 eyes) of the examined eyes showed a “high-backed chair” pattern. The remaining 87.5% were divided into a group with a lower posterior spinous hump, corresponding to that of the previous group (62.5% of the examined eyes), and a group with a more pronounced backscatter from the side of Descemet’s membrane on the densitogram (25% of the examined eyes).

In the last age group (80–89 years) of men with FECD, densitograms of 8 eyes were analyzed. When analyzing the data in this subgroup, no densitogram with a “high-backed chair” pattern was detected, unlike the corresponding group in women with FECD. In 25% (2 eyes), a low posterior spicule was observed, and in the remaining 75%, a strongly pronounced backscatter from the Descemet’s membrane, corresponding to a “hammock” densitogram pattern.

**Figure 1:**
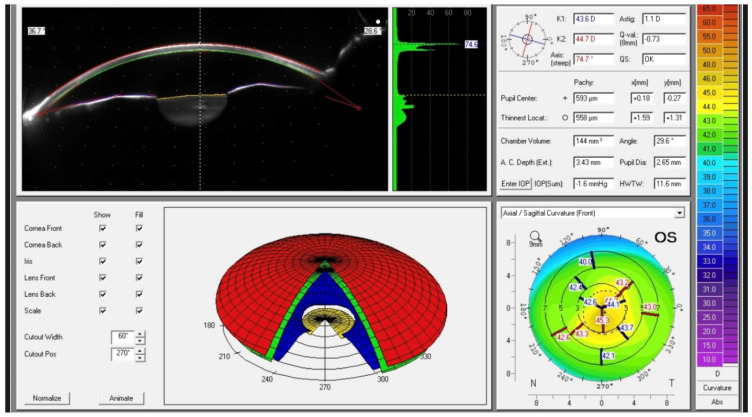
Appearance of a “two-humped camel.

As a summary of the results obtained from corneal tomography, we can say that a significant increase in the backscatter of light was observed in all layers of the cornea, with the strongest in the anterior layer, followed by the central and posterior layers. Also, an increase in densitometry values was observed with advancing age in both controls and patients with FECD, with the latter being more pronounced. From the results obtained, if we compare the progression between women and men with FECD by age, it was striking that it was more pronounced in men. When analyzing the densitograms, it was also striking that with advancing age, a posterior peak gradually appeared from a “high-backed chair” pattern, corresponding to the damaged Descemet’s membrane (DM), as this peak increased and the densitogram took on the appearance of a “two-humped camel” (Figure 1). This change was again more pronounced in men.

## 4. Discussion

The present findings should be interpreted in light of several methodological considerations. First, the inclusion of both eyes may have introduced inter-eye correlation bias. Second, subgroup sizes, particularly in male cohorts, were relatively small and may have limited the statistical power. Third, some peripheral densitometry values were unexpectedly higher in controls than in FECD eyes in selected age groups. This likely reflects age-related physiological peripheral backscatter, variability related to small subgroup sample sizes, and the known increase in peripheral corneal scatter in elderly healthy subjects. These findings emphasize that densitometry changes in FECD are not uniformly distributed across all corneal zones and should be interpreted together with clinical examination and topographic parameters.

Fuchs’ endothelial corneal dystrophy (FECD) is characterized by progressive endothelial dysfunction, increased corneal hydration, and structural alterations that ultimately lead to the loss of transparency. The present study demonstrates a consistent increase in corneal densitometry values with advancing age and disease severity, with the most pronounced changes observed in the anterior corneal layer and in the peripheral zones (6–12 mm). These findings are largely in agreement with previously published data, confirming the diagnostic value of Scheimpflug-based densitometry in FECD [8].

Several authors have reported that corneal densitometry is significantly elevated in patients with FECD compared to healthy controls, reflecting increased light backscatter due to stromal edema and extracellular matrix alterations. For instance, studies by van Shah et al. (2022) and Schaub et al. (2017) demonstrated that densitometry values increase progressively with disease severity, particularly in the posterior cornea, corresponding to Descemet’s membrane changes and guttae formation [9,10]. In our study, although increased backscatter was observed in all layers, the highest values were consistently found in the anterior layer.

The zonal distribution of densitometry values in our cohort—lowest in the 2–6 mm zone and highest in the periphery (10–12 mm)—is consistent with the results reported by Schaub et al., who found that peripheral corneal zones exhibit higher baseline backscatter even in healthy individuals, with a more pronounced increase in FECD [10]. Similarly, Otri et al. reported that densitometric changes are not limited to the central cornea but extend toward the periphery as the disease progresses, which supports our observation of significant differences, particularly in the 6–10 mm and 10–12 mm zones [11].

An important finding in this study is the age-related progression of densitometric values, especially when comparing the youngest (50–59 years) and oldest (80–89 years) subgroups. This trend is well documented in the literature. Cleynenbreugel et al. demonstrated that corneal backscatter increases physiologically with age, even in healthy subjects, due to changes in collagen organization and hydration [12]. However, in FECD patients, this increase is significantly accelerated, as also observed in our results. The progressive increase in backscatter from Descemet’s membrane, reflected in the transition from a “high-backed chair” to a “hammock” densitogram pattern, is consistent with the structural changes described histopathologically, including thickening of Descemet’s membrane and accumulation of guttae (Adamis et al., 1993) [13].

Another notable aspect of our findings is the gender-related difference in disease progression. Although FECD is more prevalent in women, our results suggest that densitometric progression may be more pronounced in men. This observation has been less frequently discussed in the literature, but some genetic studies, such as those involving TCF4 repeat expansions (Wieben et al., 2012), suggest variability in phenotypic expression that could potentially explain such differences [14]. Further investigation is warranted to clarify this aspect.

The qualitative densitogram analysis in our study also aligns with previously described patterns. The “high-backed chair” configuration in early stages and the “hammock” pattern in advanced FECD have been reported by Patel et al. as characteristic indicators of disease progression [15]. Our data confirm that the appearance of a posterior hump corresponding to Descemet’s membrane becomes more prominent with age and disease severity, supporting the utility of densitograms as a non-invasive biomarker.

## 5. Conclusions

In conclusion, the present study suggests that corneal densitometry obtained via Pentacam Scheimpflug tomography provides valuable quantitative and qualitative information for the assessment of FECD. The observed increase in backscatter across all layers, its age-related progression, and the characteristic densitogram patterns are consistent with existing literature. The combined analysis of topographic and densitometric parameters enhances early detection and monitoring of disease progression, which is essential for timely therapeutic decision-making.

## Figures and Tables

**Table 1:** Distribution of patients by age, gender, and health status.

Age (Years)	Patients		Total	Patients with FECD			Without FECD		
	From Them:			From Them:		Total	From Them:		Total
	Female	Male		Female	Male		Female	Male	
**50–59**	11	0	11	7	0	7	4	0	4
**60–69**	13	9	22	7	4	11	6	5	11
**70–79**	17	15	32	9	8	17	8	7	15
**80–89**	17	7	24	8	4	12	9	3	12
**Total**	58	31	89	31	16	47	27	15	42

**Table 2:** Comparison of the mean values and standard deviation of corneal densitometry in women with FECD and controls from the first age subgroup (50–59 years).

Zone	Female—FECD	Female—Controls	*p*-Value
	50–59_Γ_. ± SD	50–59_Γ_. ± SD	(Unpaired *t*-Test)
**0–2 mm**			
Anterior	23.888 ± 1.5560	24.550 ± 1.0607	t = −1.204///*p* = 0.268
Central	15.425 ± 0.8430	16.650 ± 0.4950	t = −4.110///*p* = 0.005
Posterior	11.175 ± 0.9285	12.000 ± 0.4243	t = −2.513///*p* = 0.040
Total	17.200 ± 1.3512	17.750 ± 0.6364	t = −1.151///*p* = 0.287
**2–6 mm**			
Anterior	21.688 ± 1.2449	21.700 ± 0.7071	t = −0.028///*p* = 0.978
Central	14.475 ± 0.9794	14.800 ± 0.4243	t = −0.939///*p* = 0.379
Posterior	11.025 ± 1.0674	10.950 ± 0.2121	t = 0.199///*p* = 0.848
Total	15.725 ± 0.9192	15.800 ± 0.4243	t = −0.231///*p* = 0.824
**6–10 mm**			
Anterior	27.838 ± 5.3348	20.350 ± 1.2021	t = 3.970///*p* = 0.005
Central	21.000 ± 3.9464	15.400 ± 0.7071	t = 4.014///*p* = 0.005
Posterior	17.288 ± 2.8211	13.550 ± 0.7778	t = 3.747///*p* = 0.007
Total	22.037 ± 3.9663	16.450 ± 0.9192	t = 3.985///*p* = 0.005
**10–12 mm**			
Anterior	35.613 ± 7.7665	22.150 ± 2.7577	t = 4.903///*p* = 0.002
Central	26.138 ± 3.7804	18.450 ± 1.6263	t = 5.752///*p* = 0.001
Posterior	23.363 ± 2.4784	17.450 ± 0.7778	t = 6.747///*p* < 0.001
Total	28.363 ± 4.5356	19.350 ± 1.2021	t = 5.620///*p* = 0.001
**Total**			
Anterior	26.463 ± 3.2802	21.800 ± 1.2728	t = 4.020///*p* = 0.005
Central	18.763 ± 2.1692	15.900 ± 0.7071	t = 3.732///*p* = 0.007
Posterior	15.163 ± 1.6106	13.100 ± 0.2828	t = 3.622///*p* = 0.008
Total	20.138 ± 2.2659	16.900 ± 0.7071	t = 4.041///*p* = 0.005

**Table 3:** Comparison of mean values and standard deviation of corneal densitometry in women with FECD and controls from the second sub-age group (60–69 years).

Zone	Female—FECD	Female—Controls	*p*-Value
	60–69_Γ_. ± SD	60–69_Γ_. ± SD	(Unpaired *t*-Test)
**0–2 mm**			
Anterior	24.263 ± 3.9151	22.283 ± 2.2895	t = 1.430///*p* = 0.196
Central	15.925 ± 1.9869	14.850 ± 1.4598	t = 1.530///*p* = 0.170
Posterior	12.400 ± 0.8536	10.500 ± 1.0373	t = 6.296///*p* < 0.001
Total	17.525 ± 1.7982	15.900 ± 1.3266	t = 2.556///*p* = 0.038
**2–6 mm**			
Anterior	22.025 ± 3.6842	21.067 ± 2.2941	t = 0.735///*p* = 0.486
Central	14.763 ± 1.9033	14.583 ± 1.5651	t = 0.267///*p* = 0.797
Posterior	11.813 ± 1.0535	11.233 ± 1.2127	t = 1.556///*p* = 0.164
Total	16.188 ± 1.9874	15.617 ± 1.2828	t = 0.812///*p* = 0.444
**6–10 mm**			
Anterior	27.675 ± 3.6339	29.017 ± 6.6394	t = −1.045///*p* = 0.331
Central	20.950 ± 1.5955	22.000 ± 3.5558	t = −1.861///*p* = 0.105
Posterior	17.713 ± 1.7382	19.817 ± 2.3761	t = −3.425///*p* = 0.011
Total	22.150 ± 2.3183	23.933 ± 4.5781	t = −2.175///*p* = 0.066
**10–12 mm**			
Anterior	35.375 ± 5.2825	34.033 ± 7.2987	t = 0.719///*p* = 0.496
Central	26.575 ± 3.3397	27.217 ± 2.8089	t = −0.544///*p* = 0.604
Posterior	24.075 ± 4.4577	25.633 ± 2.5649	t = −0.989///*p* = 0.356
Total	28.675 ± 3.5596	29.517 ± 3.1815	t = −0.669///*p* = 0.525
**Total**			
Anterior	26.525 ± 3.3435	26.183 ± 3.5414	t = 0.289///*p* = 0.781
Central	18.913 ± 1.8114	19.050 ± 1.7774	t = −2.15///*p* = 0.836
Posterior	15.825 ± 1.3583	16.217 ± 1.4261	t = −0.816///*p* = 0.441
Total	20.550 ± 1.8509	20.467 ± 2.0146	t = 0.127///*p* = 0.903

**Table 4:** Comparison of mean values and standard deviation of corneal densitometry in women with FECD and controls from the third sub-age group (70–79 years).

Zone	Female—FECD	Female—Controls	*p*-Value
	70–79_Γ_. ± SD	70–79_Γ_. ± SD	(Unpaired *t*-Test)
**0–2 mm**			
Anterior	23.725 ± 1.9101	23.912 ± 1.3109	t = −0.392///*p* = 0.701
Central	15.994 ± 1.2119	16.200 ± 1.1796	t = −0.681///*p* = 0.506
Posterior	12.069 ± 1.5785	11.213 ± 1.0092	t = 2.169///*p* = 0.047
Total	17.275 ± 1.4017	17.113 ± 1.0288	t = 0.462///*p* = 0.650
**2–6 mm**			
Anterior	26.213 ± 9.2993	22.750 ± 1.7800	t = 1.489///*p* = 0.157
Central	16.506 ± 3.4358	15.700 ± 1.6475	t = 0.939///*p* = 0.363
Posterior	12.575 ± 2.7017	11.575 ± 1.3318	t = 1.481///*p* = 0.159
Total	17.719 ± 3.7050	16.662 ± 1.4928	t = 1.141///*p* = 0.272
**6–10 mm**			
Anterior	43.238 ± 13.1067	40.900 ± 8.6277	t = 0.713///*p* = 0.487
Central	31.106 ± 8.8691	29.538 ± 3.3628	t = 0.707///*p* = 0.490
Posterior	21.944 ± 4.5850	22.800 ± 2.5383	t = −0.747///*p* = 0.467
Total	32.050 ± 8.7241	31.688 ± 3.6018	t = 0.166///*p* = 0.870
**10–12 mm**			
Anterior	50.075 ± 9.3845	49.300 ± 17.476	t = 0.370///*p* = 0.717
Central	31.006 ± 4.3840	31.588 ± 6.4780	t = −0.531///*p* = 0.603
Posterior	24.031 ± 3.2224	27.600 ± 4.0043	t = −4.430///*p* < 0.001
Total	35.063 ± 4.7118	36.163 ± 8.8880	t = − 0.934///*p* = 0.365
**Total**			
Anterior	33.833 ± 6.6833	33.625 ± 4.3107	t = 0.123///*p* = 0.903
Central	23.631 ± 4.4670	22.888 ± 2.1820	t = 0.666///*p* = 0.516
Posterior	17.438 ± 2.8059	17.763 ± 1.6501	t = −0.464///*p* = 0.649
Total	25.175 ± 4.5714	24.700 ± 2.6005	t = 0.416///*p* = 0.684

**Table 5:** Comparison of the mean values and standard deviation of corneal densitometry in women with FECD and controls from the fourth sub-age group (80–89 years).

Zone	Female FECD	Female—Controls	*p*-Value
	80–89_Γ_. ± SD	80–89_Γ_. ± SD	(Unpaired *t*-Test)
**0–2 mm**			
Anterior	27.629 ± 2.8256	23.420 ± 2.4485	t = 5.573///*p* < 0.001
Central	17.493 ± 2.0694	15.970 ± 1.1672	t = 2.754///*p* = 0.016
Posterior	14.221 ± 5.6251	11.470 ± 1.3458	t = 1.830///*p* = 0.090
Total	19.786 ± 3.3094	16.960 ± 1.4924	t = 3.195///*p* = 0.007
**2–6 mm**			
Anterior	25.621 ± 2.6318	21.890 ± 1.7110	t = 5.305///*p* < 0.001
Central	16.636 ± 1.8362	15.290 ± 1.1976	t = 2.742///*p* = 0.017
Posterior	13.436 ± 3.8793	11.670 ± 1.6707	t = 1.703///*p* = 0.112
Total	18.550 ± 2.4140	16.290 ± 1.3203	t = 3.503///*p* = 0.004
**6–10 mm**			
Anterior	37.336 ±10.4507	36.400 ± 13.3930	t = 0.335///*p* = 0.743
Central	26.536 ± 6.6736	26.680 ± 9.1892	t = −0.081///*p* = 0.937
Posterior	21.264 ± 5.0363	19.490 ± 4.9020	t = 1.318///*p* = 0.210
Total	28.429 ± 7.1755	27.730 ± 8.7215	t = 0.364///*p* = 0.722
**10–12 mm**			
Anterior	39.914 ±12.1492	48.850 ± 21.6259	t = −2.752///*p* = 0.016
Central	28.407 ± 7.0336	29.040 ± 9.6765	t = −0.337///*p* = 0.742
Posterior	24.250 ± 5.2721	21.880 ± 5.3876	t = 1.682///*p* = 0.116
Total	30.586 ± 8.0551	33.250 ± 11.8778	t = −1.238///*p* = 0.238
**Total**			
Anterior	32.271 ± 6.0982	30.940 ± 7.5515	t = 0.817///*p* = 0.429
Central	21.964 ± 3.8767	21.240 ± 4.6407	t = 0.699///*p* = 0.497
Posterior	17.900 ± 4.0785	15.770 ± 2.9315	t = 1.954///*p* = 0.073
Total	24.057 ± 4.2583	22.660 ± 4.8454	t = 1.228///*p* = 0.241

**Table 6:** Comparison of mean values and standard deviation of corneal densitometry in women with FECD from the first and fourth sub-age groups (50–59 years; 80–89 years).

Zone	Females—FECD	Female—FECD	*p*-Value
	50–59_Γ_. ± SD	80–89_Γ_. ± SD	(Unpaired *t*-Test)
**0–2 mm**			
Anterior	23.888 ± 1.5560	27.629 ± 2.8256	t = 4.953///*p* < 0.001
Central	15.425 ± 0.8430	17.493 ± 2.0694	t = 3.739///*p* = 0.002
Posterior	11.175 ± 0.9285	14.221 ± 5.6251	t = 2.026///*p* = 0.064
Total	17.200 ± 1.3512	19.786 ± 3.3094	t = 2.923///*p* = 0.012
**2–6 mm**			
Anterior	21.688 ± 1.2449	25.621 ± 2.6318	t = 5.592///*p* < 0.001
Central	14.475 ± 0.9794	16.636 ± 1.8362	t = 4.403///*p* = 0.001
Posterior	11.025 ± 1.0674	13.436 ± 3.8793	t = 2.325///*p* = 0.037
Total	15.725 ± 0.9192	18.550 ± 2.4140	t = 4.379///*p* = 0.001
**6–10 mm**			
Anterior	27.838 ± 5.3348	37.336 ±10.4507	t = 3.400///*p* = 0.005
Central	21.000 ± 3.9464	26.536 ± 6.6736	t = 3.104///*p* = 0.008
Posterior	17.288 ± 2.8211	21.264 ± 5.0363	t = 2.954///*p* = 0.011
Total	22.037 ± 3.9663	28.429 ± 7.1755	t = 3.333///*p* = 0.005
**10–12 mm**			
Anterior	35.613 ± 7.7665	39.914 ±12.1492	t = 1.325///*p* = 0.208
Central	26.138 ± 3.7804	28.407 ± 7.0336	t = 1.207///*p* = 0.249
Posterior	23.363 ± 2.4784	24.250 ± 5.2721	t = 0.630///*p* = 0.540
Total	28.363 ± 4.5356	30.586 ± 8.0551	t = 1.032///*p* = 0.321
**Total**			
Anterior	26.463 ± 3.2802	32.271 ± 6.0982	t = 3.564///*p* = 0.003
Central	18.763 ± 2.1692	21.964 ± 3.8767	t = 3.090///*p* = 0.009
Posterior	15.163 ± 1.6106	17.900 ± 4.0785	t = 2.511///*p* = 0.026
Total	20.138 ± 2.2659	24.057 ± 4.2583	t = 3.444///*p* = 0.004

**Table 7:** Comparison of the mean values and standard deviation of corneal densitometry in men with FECD and controls from the first sub-age group (60–69 years).

Zone	Male FECD	Male—Controls	*p*-Value
	60–69_Γ_. ± SD	60–69_Γ_. ± SD	(Unpaired *t*-Test)
**0–2 mm**			
Anterior	24.200 ± 0.5164	23.450 ± 2.1131	t = 2.905///*p* = 0.62
Central	16.375 ± 1.4796	15.540 ± 1.4315	t = 1.129///*p* = 0.341
Posterior	11.750 ± 0.9110	11.220 ± 1.2318	t = 1.164///*p* = 0.329
Total	17.450 ± 0.9037	16.740 ± 1.4531	t = 1.571///*p* = 0.214
**2–6 mm**			
Anterior	22.175 ± 0.3500	21.930 ± 1.9675	t = 1.400///*p* = 0.256
Central	15.225 ± 0.7848	14.840 ± 1.1047	t = 0.981///*p* = 0.399
Posterior	11.425 ± 0.4349	11.320 ± 1.0401	t = 0.483///*p* = 0.662
Total	16.275 ± 0.4031	16.040 ± 1.2756	t = 1.166///*p* = 0.328
**6–10 mm**			
Anterior	23.400 ± 2.5020	35.670 ± 6.1932	t = −9.808///*p* = 0.002
Central	18.275 ± 1.7193	25.960 ± 4.8553	t = −8.940///*p* = 0.003
Posterior	14.875 ± 0.9605	19.640 ± 3.2325	t = −9.922///*p* = 0.002
Total	18.850 ± 1.6462	27.120 ± 4.4743	t = −10.047///*p* = 0.002
**10–12 mm**			
Anterior	31.225 ± 6.3731	44.830 ± 13.9989	t = −4.270///*p* = 0.024
Central	22.650 ± 3.7969	30.120 ± 6.2907	t = −3.935///*p* = 0.029
Posterior	20.500 ± 2.3791	24.590 ± 36.3640	t = −3.438///*p* = 0.041
Total	24.775 ± 4.1210	34.450 ± 6.6958	t = −4.695///*p* = 0.018
**Total**			
Anterior	24.425 ± 1.0210	30.910 ± 3.3811	t = −12.703///*p* = 0.001
Central	17.675 ± 0.6702	21.200 ± 2.6829	t = −10.519///*p* = 0.002
Posterior	14.150 ± 0.8583	16.580 ± 2.1776	t = −5.662///*p* = 0.011
Total	18.750 ± 0.7895	22.890 ± 2.5826	t = −10.487///*p* = 0.002

**Table 8:** Comparison of mean values and standard deviation of corneal densitometry in men with FECD and controls from the second sub-age group (70–79 years).

Zone	Male—FECD	Male—Controls	*p*-Value
	70–79_Γ_. ± SD	70–79_Γ_. ± SD	(Unpaired *t*-Test)
**0–2 mm**			
Anterior	27.238 ± 3.5557	25.967 ± 2.9095	t = 1.011///*p* = 0.346
Central	17.725 ± 0.6296	16.444 ± 0.9606	t = 5.755///*p* = 0.001
Posterior	13.050 ± 0.8816	11.733 ± 0.8902	t = 4.226///*p* = 0.004
Total	19.325 ± 1.4400	18.044 ± 1.4266	t = 2.516///*p* = 0.040
**2–6 mm**			
Anterior	28.788 ± 3.2726	25.089 ± 2.9105	t = 3.197///*p* = 0.015
Central	19.700 ± 2.8173	16.356 ± 0.9153	t = 3.357///*p* = 0.012
Posterior	14.013 ± 0.7900	12.156 ± 0.8156	t = 6.647///*p* < 0.001
Total	20.838 ± 1.9449	17.856 ± 1.4152	t = 4.336///*p* = 0.003
**6–10 mm**			
Anterior	45.900 ± 15.9006	43.100 ± 7.4108	t = 0.498///*p* = 0.634
Central	32.400 ± 8.1689	29.000 ± 6.6295	t = 1.177///*p* = 0.278
Posterior	23.350 ± 4.8744	20.533 ± 2.8031	t = 1.635///*p* = 0.146
Total	34.313 ± 8.6062	29.800 ± 6.3849	t = 1.483///*p* = 0.182
**10–12 mm**			
Anterior	48.900 ± 13.4498	59.256 ± 13.6491	t = −2.178///*p* = 0.066
Central	28.650 ± 4.6022	33.522 ± 11.5335	t = −2.994///*p* = 0.020
Posterior	24.488 ± 4.0825	25.589 ± 6.7245	t = −0.763///*p* = 0.470
Total	34.012 ± 6.9501	38.333± 11.7546	t = −1.758///*p* = 0.122
**Total**			
Anterior	37.788 ± 6.7198	35.167 ± 6.3618	t = 1.103///*p* = 0.306
Central	25.050 ± 4.0032	22.967 ± 3.1421	t = 1.472///*p* = 0.185
Posterior	18.575 ± 2.3789	16.756 ± 1.4178	t = 2.163///*p* = 0.067
Total	27.162 ± 4.0415	25.356 ± 2.4895	t = 1.264///*p* = 0.247

**Table 9:** Comparison of the mean values and standard deviation of corneal densitometry in men with FECD and controls from the third sub-age group (80–89 years).

Zone	Male—FECD	Male—Controls	*p*-Value
	80–89_Γ_. ± SD	80–89_Γ_. ± SD	(Unpaired *t*-Test)
**0–2 mm**			
Anterior	36.417 ± 12.2405	37.575 ± 24.0224	t = −0.232///*p* = 0.826
Central	20.433 ± 4.3029	20.075 ± 3.7411	t = 0.204///*p* = 0.846
Posterior	17.650 ± 5.6589	13.225 ± 2.3343	t = 1.915///*p* = 0.114
Total	24.850 ± 7.3878	23.650 ± 9.9848	t = 0.398///*p* = 0.707
**2–6 mm**			
Anterior	30.050 ± 6.3576	34.600 ± 17.8705	t = −1.753///*p* = 0.140
Central	18.483 ± 2.6362	19.975 ± 2.6924	t = −1.386///*p* = 0.224
Posterior	14.500 ± 1.9026	13.650 ± 1.4708	t = 1.094///*p* = 0.324
Total	21.000 ± 3.5777	22.750 ± 7.3178	t = −1.198///*p* = 0.285
**6–10 mm**			
Anterior	40.217 ± 9.2813	53.900 ± 10.1551	t = −3.611///*p* = 0.015
Central	28.267 ± 5.6864	35.450 ± 4.6522	t = −3.094///*p* = 0.027
Posterior	21.017 ± 3.6439	23.625 ± 2.5065	t = −1.753///*p* = 0.140
Total	30.000 ± 6.1116	37.650 ± 5.6300	t = −3.066///*p* = 0.028
**10–12 mm**			
Anterior	48.983 ± 7.1876	63.700 ± 18.7679	t = −5.015///*p* = 0.004
Central	28.817 ± 2.5810	35.900 ± 9.3652	t = −6.722///*p* = 0.001
Posterior	23.300 ± 2.7357	25.250 ± 1.5264	t = −1.746///*p* = 0.141
Total	33.717 ± 3.2382	41.600 ± 9.8718	t = −5.963///*p* = 0.002
**Total**			
Anterior	37.600 ± 7.0759	45.325 ± 13.7510	t = −2.674///*p* = 0.044
Central	23.783 ± 3.7129	27.350 ± 3.5726	t = −2.337///*p* = 0.067
Posterior	18.667 ± 3.0618	18.650 ± 1.3892	t = 0.013///*p* = 0.990
Total	26.700 ± 4.5233	30.450 ± 6.0973	t = −2.031///*p* = 0.098

**Table 10:** Comparison of mean values and standard deviation of corneal densitometry in men with FECD from the third and first sub-age groups (80–89 years; 60–69 years).

Zone	Male—FECD	Male—FECD	*p*-Value
	80–89_Γ_. ± SD	60–69_Γ_. ± SD	(Unpaired *t*-Test)
**0–2 mm**			
Anterior	36.417 ± 12.2405	24.200 ± 0.5164	t = 2.445///*p* = 0.050
Central	20.433 ± 4.3029	16.375 ± 1.4796	t = 2.310///*p* = 0.065
Posterior	17.650 ± 5.6589	11.750 ± 0.9110	t = 2.554///*p* = 0.047
Total	24.850 ± 7.3878	17.450 ± 0.9037	t = 2.454///*p* = 0.054
**2–6 mm**			
Anterior	30.050 ± 6.3576	22.175 ± 0.3500	t = 3.032///*p* = 0.029
Central	18.483 ± 2.6362	15.225 ± 0.7848	t = 3.028///*p* = 0.029
Posterior	14.500 ± 1.9026	11.425 ± 0.4349	t = 3.959///*p* = 0.011
Total	21.000 ± 3.5777	16.275 ± 0.4031	t = 3.235///*p* = 0.023
**6–10 mm**			
Anterior	40.217 ± 9.2813	23.400 ± 2.5020	t = 4.438///*p* = 0.007
Central	28.267 ± 5.6864	18.275 ± 1.7193	t = 4.304///*p* = 0.008
Posterior	21.017 ± 3.6439	14.875 ± 0.9605	t = 4.129///*p* = 0.009
Total	30.000 ± 6.1116	18.850 ± 1.6462	t = 4.469///*p* = 0.007
**10–12 mm**			
Anterior	48.983 ± 7.1876	31.225 ± 6.3731	t = 6.052///*p* = 0.002
Central	28.817 ± 2.5810	22.650 ± 3.7969	t = 5.852///*p* = 0.002
Posterior	23.300 ± 2.7357	20.500 ± 2.3791	t = 2.507///*p* = 0.054
Total	33.717 ± 3.2382	24.775 ± 4.1210	t = 6.764///*p* = 0.001
**Total**			
Anterior	37.600 ± 7.0759	24.425 ± 1.0210	t = 4.561///*p* = 0.006
Central	23.783 ± 3.7129	17.675 ± 0.6702	t = 4.030///*p* = 0.010
Posterior	18.667 ± 3.0618	14.150 ± 0.8583	t = 3.613///*p* = 0.015
Total	26.700 ± 4.5233	18.750 ± 0.7895	t = 4.305///*p* = 0.008

## Data Availability

The datasets generated during and/or analyzed during the current study are available from the corresponding author on reasonable request.

## Supplementary Materials

The following supporting information can be downloaded at: https://www.mdpi.com/article/10.3390/medicina62071309/s1.
