# Supplementary material for: Modern Diagnostic Modalities for Fuchs’ Endothelial Corneal Dystrophy: A Comparative Analysis Using Scheimpflug Tomography
Source: Medicina (Kaunas). 2026 Jul 6;62(7):1309. doi: 10.3390/medicina62071309 (PMC13414369; doi:10.3390/medicina62071309)
Supplement: Supplementary file 1 [file medicina-62-01309-s001.zip › Table S1.pdf]

Table S1. Demographic distribution by age, sex, and health status, divided into two main groups: control (healthy individuals) and the group with Fuchs' endothelial corneal dystrophy (FECD). For each subgroup, the number of individuals (n) and their mean age with standard deviation are indicated, allowing comparison between females and males in different age intervals from 50 to 89 years. The results reveal a predominant participation of females (65.17% of the total), with a slightly higher mean age in males in the FECD group (75.44 years) compared to females (70.71 years), as well as a balanced distribution by age groups between controls and patients.

**Group**	**Sex**	**Age Group (years)**	**n (Individuals)**	**Mean Age ± SD (years)**
Control	Female	50–59	6	54.50 ± 2.81
		60–69	9	64.67 ± 2.96
		70–79	8	74.25 ± 3.20
		80–89	4	83.00 ± 2.45
	Male	60–69	5	65.20 ± 3.11
		70–79	6	74.83 ± 2.99
		80–89	4	84.50 ± 2.65
FECD	Female	50–59	7	55.86 ± 1.95
		60–69	10	65.10 ± 3.03
		70–79	8	74.38 ± 3.16
		80–89	6	83.67 ± 2.58
	Male	60–69	6	65.50 ± 3.08
		70–79	7	74.71 ± 3.15
		80–89	3	85.00 ± 2.65

Specular Microscopy Findings In FECD eyes, mean CD was 2162.21 ± 672.78 cells/mm², CV 34.60 ± 6.26%, HEX 62.14 ± 7.48%, CCT 559.58 ± 39.94 µm. Age-related declines in CD and HEX, with CV increases, were significant, more in males. Key comparisons in Tables 2 and 3.
