# Supplementary material for: Modern Diagnostic Modalities for Fuchs’ Endothelial Corneal Dystrophy: A Comparative Analysis Using Scheimpflug Tomography
Source: Medicina (Kaunas). 2026 Jul 6;62(7):1309. doi: 10.3390/medicina62071309 (PMC13414369; doi:10.3390/medicina62071309)
Supplement: Supplementary file 1 [file medicina-62-01309-s001.zip › Table S2.pdf]

Table S2. Specular microscopy parameters for females with Fuchs' endothelial corneal dystrophy (FECD) compared to controls, aggregated across all age groups, including metrics such as endothelial cell count (NUM), cell density (CD), average cell size (AVG), standard deviation (SD), coefficient of variation (CV), maximum and minimum cell sizes (MAX/MIN), hexagonality (HEX), and central corneal thickness (CCT). Each parameter is reported with mean values and standard deviations for both groups, along with p-values indicating statistical significance. The results demonstrate significant reductions in cell density (CD), hexagonality (HEX), and cell count (NUM) in FECD patients, coupled with increases in cell size variability (CV, AVG, SD) and corneal thickness (CCT), highlighting pronounced endothelial dysfunction in affected females compared to healthy controls.

**Parameter**	**FECD (Mean ± SD)**	**Control (Mean ± SD)**	**p-value**
NUM	145.2 ± 45.3	198.7 ± 32.1	<0.001
CD (cells/mm²)	2162 ± 673	2654 ± 412	<0.001
AVG (µm²)	478.5 ± 145.2	381.2 ± 58.4	<0.001
SD	168.3 ± 52.1	128.4 ± 38.7	0.002
CV (%)	34.6 ± 6.3	29.8 ± 4.5	0.005
MAX	912.4 ± 278.6	756.3 ± 189.2	0.031
MIN	198.7 ± 65.4	245.1 ± 72.3	0.005
HEX (%)	62.1 ± 7.5	68.4 ± 6.2	<0.001
CCT (µm)	559.6 ± 39.9	542.3 ± 35.1	0.010
