# Supplementary material for: Modern Diagnostic Modalities for Fuchs’ Endothelial Corneal Dystrophy: A Comparative Analysis Using Scheimpflug Tomography
Source: Medicina (Kaunas). 2026 Jul 6;62(7):1309. doi: 10.3390/medicina62071309 (PMC13414369; doi:10.3390/medicina62071309)
Supplement: Supplementary file 1 [file medicina-62-01309-s001.zip › Table S3.pdf]

Table S3. Specular microscopy parameters for males with Fuchs' endothelial corneal dystrophy (FECD) compared to controls, aggregated across all age groups, including metrics such as endothelial cell count (NUM), cell density (CD), average cell size (AVG), standard deviation (SD), coefficient of variation (CV), maximum and minimum cell sizes (MAX/MIN), hexagonality (HEX), and central corneal thickness (CCT). Each parameter is reported with mean values and standard deviations for both groups, along with p-values indicating statistical significance. The results demonstrate significant reductions in cell density (CD), hexagonality (HEX), and cell count (NUM) in FECD patients, coupled with increases in cell size variability (CV, AVG, SD) and corneal thickness (CCT), underscoring pronounced endothelial dysfunction in affected males compared to healthy controls, with the exception of maximum cell size (MAX), which showed no significant difference.

**Parameter**	**FECD (Mean ± SD)**	**Control (Mean ± SD)**	**p-value**
NUM	132.4 ± 48.7	192.5 ± 34.6	<0.001
CD (cells/mm²)	1987 ± 712	2589 ± 456	<0.001
AVG (µm²)	512.3 ± 162.4	392.1 ± 67.8	<0.001
SD	182.6 ± 59.3	134.7 ± 42.1	0.001
CV (%)	36.8 ± 7.1	30.2 ± 5.3	0.002
MAX	945.7 ± 301.2	789.4 ± 201.5	NS
MIN	185.4 ± 71.2	238.9 ± 78.4	0.005
HEX (%)	58.9 ± 8.2	67.1 ± 6.9	0.045
CCT (µm)	568.2 ± 42.3	548.7 ± 37.4	0.001
