# Supplementary material for: Modern Diagnostic Modalities for Fuchs’ Endothelial Corneal Dystrophy: A Comparative Analysis Using Scheimpflug Tomography
Source: Medicina (Kaunas). 2026 Jul 6;62(7):1309. doi: 10.3390/medicina62071309 (PMC13414369; doi:10.3390/medicina62071309)
Supplement: Supplementary file 1 [file medicina-62-01309-s001.zip › Table S4.pdf]

Table S4. Corneal densitometry and topographic features in patients with Fuchs' endothelial corneal dystrophy (FECD) compared to controls, including total densitometry across the 0–12 mm zone, layer-specific values (anterior, central, posterior), zone-specific values (0–2 mm and 2–6 mm), thinnest point displacement (Y-axis in mm), and isopach loss percentage. Each parameter is reported with mean values and standard deviations for both groups, along with p-values indicating statistical significance. The results demonstrate significantly elevated densitometry in all measured layers and zones for FECD patients, coupled with greater thinnest point displacement and isopach loss, underscoring increased corneal backscatter, opacity, and topographic irregularities in the diseased group compared to healthy controls.

**Parameter/Layer or Zone**	**FECD (GSU Mean ± SD)**	**Control (GSU Mean ± SD)**	**p-value**
Total Densitometry (0–12 mm)	28.8 ± 6.7	24.3 ± 4.1	<0.001
Anterior Layer	39.0 ± 12.0	25.8 ± 5.1	<0.001
Central Layer	25.0 ± 7.1	18.2 ± 3.4	<0.001
Posterior Layer	23.5 ± 6.8	16.8 ± 2.9	<0.001
Zone 0–2 mm	28.0 ± 7.2	17.4 ± 2.2	<0.001
Zone 2–6 mm	24.4 ± 6.4	17.6 ± 2.4	<0.001
Thinnest Point Displacement (Y, mm)	0.85 ± 0.32	0.62 ± 0.21	0.014
Isopach Loss (%)	68.75 (advanced)	0 (early)	<0.001
